# Comparing Attitudes Toward Different Consent Mediums: Semistructured Qualitative Study

**DOI:** 10.2196/53113

**Published:** 2024-04-30

**Authors:** Xengie Doan, Arianna Rossi, Marietjie Botes, Annika Selzer

**Affiliations:** 1 SnT University of Luxembourg Esch-sur-Alzette Luxembourg; 2 LIDER Lab DIRPOLIS Scuola Superiore Sant'Anna Pisa Italy; 3 Department of Medicine Stellenbosch University Stellenbosch South Africa; 4 Fraunhofer Institute for Secure Information Technology Darmstadt Germany; 5 ATHENE National Research Center for Applied Cybersecurity Darmstadt Germany

**Keywords:** consent, transparency, data governance, visualization, health data sharing

## Abstract

**Background:**

As consent for data sharing evolves with the digital age, plain-text consent is not the only format in which information can be presented. However, designing a good consent form is highly challenging. The addition of graphics, video, and other mediums to use can vary widely in effectiveness; and improper use can be detrimental to users.

**Objective:**

This study aims to explore the expectations and experiences of adults toward consent given in infographic, video, text, newsletter, and comic forms in a health data sharing scenario to better understand the appropriateness of different mediums and identify elements of each medium that most affect engagement with the content.

**Methods:**

We designed mock consent forms in infographic, video, text, newsletter, and comic versions. Semistructured interviews were conducted with adults who were interviewed about their expectations for consent and were then shown each consent medium and asked about engaging elements across mediums, preferences for consent mediums, and the value of document quality criteria. We transcribed and qualitatively co-coded to identify themes and perform analyses.

**Results:**

We interviewed 24 users and identified different thematic archetypes based on participant goals, such as the Trust Seeker, who considered their own understanding and trust in organizations when making decisions. The infographic was ranked first for enhancing understanding, prioritizing information, and maintaining the proper audience fit for serious consent in health data sharing scenarios. In addition, specific elements such as structure, step-by-step organization, and readability were preferred engaging elements.

**Conclusions:**

We identified archetypes to better understand user needs and elements that can be targeted to enhance user engagement with consent forms; this can help inform the design of more effective consent in the future. Overall, preferences for mediums are highly contextual, and more research should be done.

## Introduction

### Overview

Consent is a cornerstone of ethical research, allowing people to be informed about the risks and benefits of research and demonstrate their autonomy. Consent has been discussed since the Nuremberg trials and takes on a pivotal role throughout European Union (EU) regulations for data protection, such as the General Data Protection Regulation (GDPR), but there are still challenges as bioethical consent and data protection consent collide. Digital decision-making about one’s own data can be influenced or misled through interface design choices (ie, through so-called dark patterns [1-8]), while the consent experience of most European users corresponds to nagging cookie consent requests with profiling and advertisements that induce consent fatigue while trying to access a needed service [8]. Decades of research in the biomedical domain show that study participants’ consent can rarely be deemed actually informed [9], often due to the complexity of language [10] and lack of health literacy [11], as well as the lack of data literacy of the individuals [12].

Engaging individuals in a user-friendly consent experience is thus fundamental to enabling them to meaningfully and freely make decisions with a sense of satisfaction [10] and agency. Improving the readability and comprehensibility of consent notices is one aspect of this, but research is also being done to explore visual communication techniques. Current research often focuses on the effect of multimedia on understanding [11,12], which can have a varied effect based on different studies. Multimedia also spans many formats, and most studies reviewed for their effect on understanding compared 2-3 different formats [12]. The Article 29 Working Party also refers to visual design means, such as “cartoons, infographics, flowcharts,” to enhance the comprehensibility of information, and specifically to “comics/cartoons, pictograms, animations” [13]. However, they do not offer further guidance about what mediums to use and for what purpose (eg, how one might prioritize skimming, while another might be better for complex information). Therefore, we experimented with 5 different mediums of consent in this study, building on studies researching the use of a comic [14,15], video [16], infographic, and illustrated text [17], with plain text as a control [18].

In this paper, we substantially built on our previous work [19] by analyzing more mediums beyond the comic and infographic and specific engaging elements. The study presented a fictional scenario with a data trustee who would assist organizations (eg, research institutions, hospitals, etc) in finding suitable participants for clinical trials in a privacy-friendly manner. Participants were given a scenario where they were individuals who may benefit from a clinical trial organized by a hospital, so the data intermediary requested their consent to share their contact information with the hospital.

The objective of this study was to better profile user expectations and their attitudes toward different consent mediums, which included infographic, video, text, newsletter, and comic versions. We specifically analyzed how different elements of consent mediums (eg, narrative, color, and audio) affected participant engagement to survey the different affordances of each medium. Each medium has its own strengths and weaknesses in representing various kinds of information and can achieve various informational goals (eg, the video is low effort but can be skimmed, while the text can be skimmed but boring) [20]. As we intended to understand whether there are benefits to using one medium over another and why participants would prefer different mediums, we compared multiple mediums in this study based on semistructured interviews and dived into participant motivations, expectations, and experiences.

The results hinted at diverse goals among participants. We also identified the elements of document design that make the information concise, structured, and appropriate for the audience. We also found a large influence of context (eg, cookie consent or consent with different trusted institutions) on participant perceptions and expectations. Thus, we offer recommendations on how to better design consent documents to address different general participant profiles using layering and to engage the audience more effectively with a suitable medium. This has a pivotal role in the digital health data sharing space to give more effective transparency to participants who are deciding whether to share sensitive data. Our results can be leveraged by designers of digital consent experiences for more efficient multimedia use.

### Background

#### Consent and Transparency

The European data strategy [21,22] aims to create a single market for data to allow for the free flow of data to benefit businesses, research, and public administrations within the EU. It is built on the GDPR, which aims to give users more control over their personal data.

Informed consent (IC) is a legal requirement specified in the GDPR as “freely given, specific, informed and unambiguous” (article 4(11)); easily withdrawn (article 7(3)); presented in an intelligible and easily accessible form using clear and plain language (article 7(2)); explicitly given for biomedical and genome data categorized as sensitive data (article 9); transparent in terms of completeness, comprehensibility, and accessibility of the information disclosures (articles 12, 13, and 14); and compliant with the principles of data protection by design and by default (article 25) [23]. IC requires user-centric design elements in consent to help achieve the general principle of transparency, which encompasses the “quality, accessibility, and comprehensibility of the information” [14]. The GDPR also contains obligations for “transparency by design” wherein privacy and consent notices should be purposefully designed to adequately inform the intended audience [24]. In addition, the GDPR also refers to other visual design methods like comics, videos, and infographics.

However, most existing informed decision-making solutions fail to reconcile theoretical demands with actual transparency. Conventional data privacy communication is characterized by lengthy, off-putting walls of complex jargon that impact the readability, comprehensibility, navigability, and memorability of information [20]. In addition, it is often standard, vague, or boilerplate instead of customized to the different needs and abilities of the intended audiences [25] and the type of data and processing activity. Reaching beyond plain language, in the last few years, there has been a renewed attention (and quite some experimentation) toward legal document design criteria [26] that more holistically relate to the language, writer-reader relationship, information design, and content.

#### Profiling User Needs Using Archetypes

Human-computer interaction research has used the persona technique (wherein imaginary users are assigned different profiles or personas with different goals and personalities based on demographic data) to better understand different users and needs and design suitable solutions [27]. However, it is a lengthy process that is often used for designing IT systems, not the consent processes. User need assessments have been conducted in relation to different demographics in health studies, but rather than focusing on the IC aspect, they focus on the health symptoms and how to address specific health-related needs [18,28,29]. Beyond health-related needs, we are interested more broadly in how the general adult population would interact with consent process to share information for downstream health reasons and what elements would be engaging when making informed decisions. This aspect has not been studied, to the best of our knowledge, but would be important for understanding how to strategically create effective information disclosures. Thus, we wanted to create archetypes, which capture general profiles, instead of personas, which are representations of imaginary individuals with specific population characteristics.

#### Multimedia Tools and Engagement With Digital Consent

The digitalization of data collection and use authorization allows for multimedia tools to be used during the consent process, which can have a positive outcome for participants. Overall, a systematic review of multimedia consent with videos, interactive programs, so on for surgical procedures found increased patient satisfaction for usability and informational availability [30]. However, for clinical trial consent, videos did not improve understanding [31]. Diving into the reasons that multimedia consent may be preferred to conventional text, one study compared animated videos, slideshows with voice-overs, comics, and text consent for medical practices and found that a dual-channel approach combining audio with visuals helped participant understanding [31]. This study supported older research showing that repetition of information using different multimedia means increases retention [32]. However, the specific elements of videos, comics, and text that contributed to effective communication in more general health consent were not studied—a gap that we intend to bridge with our work.

Even in other domains, studies strive to understand how to achieve effective communication of complex information by analyzing participant engagement, understanding, and recall of the information [33]. In the study by Wang et al [33], engagement refers to the time spent and fun experienced reading a form; and infographics, illustrated text, and data comics of complex economic data were tested. They found that students from different countries (aged from 18 to 35 years) preferred data comics, as they enable the greatest understanding, engagement, and enjoyment of all mediums, while the infographic performed best in esthetics and exploration, and the illustrated text performed the worst. As similar studies had not been performed on consent forms in a health scenario, we sought to study engagement as a factor of effective communication, as it might help understand what gains and retains attention within a complex digital attention economy.

Traditionally, engagement studies in biomedical consent refer to patient engagement with the research or biomedical process. Such engagement refers to participants interacting with the results of a study, updating information, or changing consent [34-36]. However, we are interested in participant motivations to consume the information in a consent form and give their initial and continued attention to a conventionally tedious process while competing in an attention economy [37,38]. Can consent forms be interesting and attention-grabbing?

### Research Questions

The previous section has gathered evidence about the interplay between GDPR transparency requirements in data protection, the use of archetypes, and multimedia tools to enhance the experience. However, we lack an understanding of user needs, the impacts of different mediums on the user experience, and user engagement. The research questions that this study sought to answer are shown in Textbox 1.

**Research questions.**

1. What kind of goal-oriented archetypes can be created to better understand participant needs for consent?
2. Across the 5 analyzed mediums (ie, infographic, video, text, newsletter, and comic forms), what were the participant rankings of different engaging elements?
3. After exposure to the consent mediums, we asked the following questions:
  - What were the participants’ rankings of consent mediums?
  - What elements reportedly influence their preference for mediums?
  - What document quality criteria concerning language, design, content, and relationship with the reader did participants value?

## Methods

### Overview

**Figure 1:**
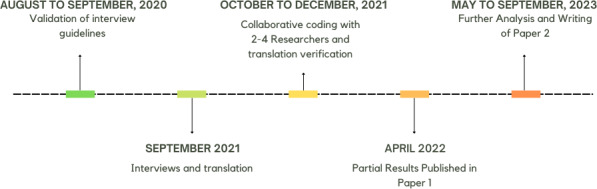
A time line of key activities and elaboration of results.

AS carried out 24 semistructured interviews in September 2021 in Germany (Figure 1). We created an interview guideline (Multimedia Appendix 1), which was validated with 3 potential participants to ensure clarity, comprehensibility, and precision of the questions.

### Recruitment

We searched for 24 participants by word of mouth. The demographic included adults from a cross-section of the German adult population by age, sex, and education level (Table 1).

**Table 1:** Participant demographics (N=24).

Characteristics		Participants, n (%)
**Age range (y)**		
	18-30	8 (33)
	31-55	8 (33)
	56-90	8 (33)
**Sex**		
	Male	12 (50)
	Female	12 (50)
**Highest degree**		
	School leaving or apprenticeship	12 (50)
	College or university	12 (50)

The sample size and participant characteristics were based on a systemic review of unbiased citizens’ juries for health policies [39]. Within the age ranges, there was an equal distribution of men and women with the highest degree obtained. All participants were native German speakers and lived in Germany, and the interviews took an average of 60 to 75 minutes.

### Study Material

**Figure 2:**
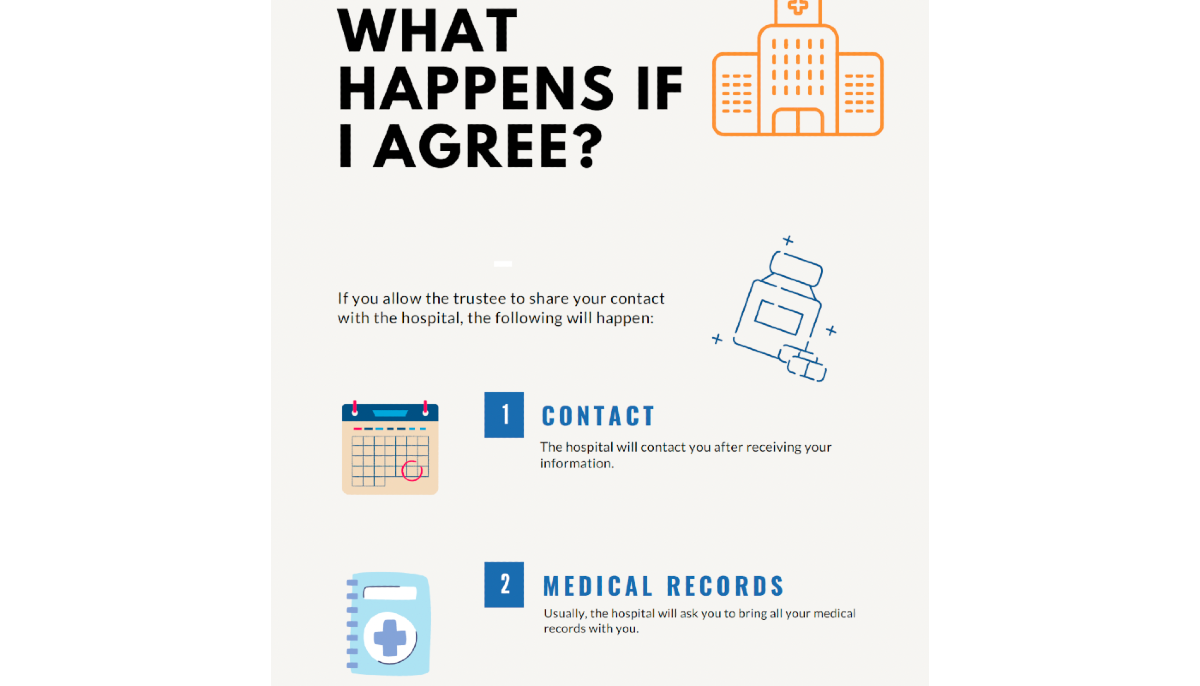
A translated section of the infographic study material designed with a step-by-step format, color, and structured sections.

**Figure 3:**
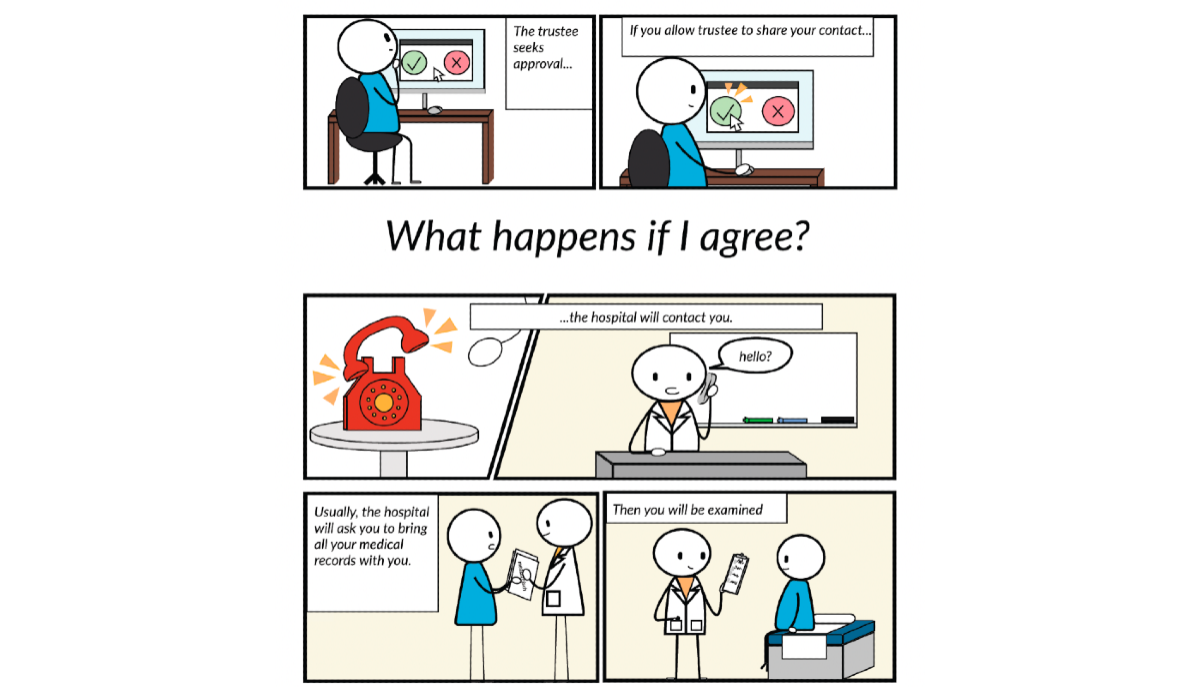
A translated section of the comic study material designed with a story and color.

**Figure 4:**
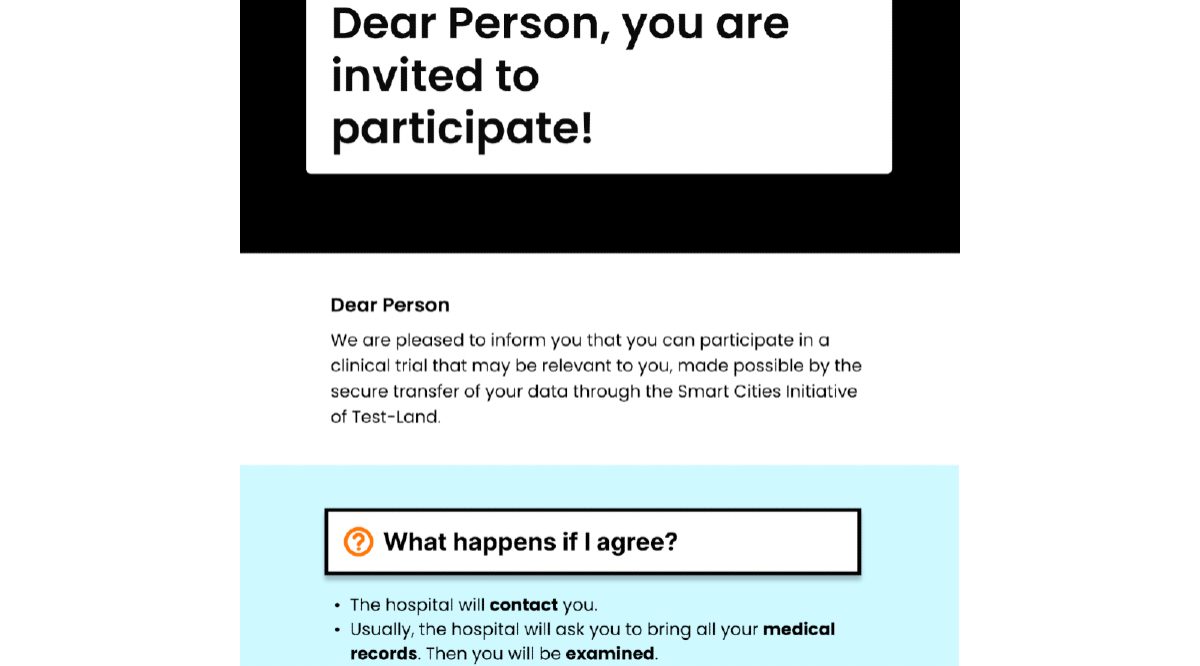
A translated section of the newsletter study material designed with an open format, color, and structured sections.

AS created an example plain text document that asked for consent for the transfer of personal data from an intermediation service to another organization, a hospital. On the basis of the plain text, XD designed 4 additional variations in different mediums: an infographic, a comic, a newsletter, and a video. These 4 variations only included the subsection “What happens if you agree?” of the consent form. All 5 consent forms (ie, plain text, infographic, comic, newsletter, and video forms) differed in design, but the core consent text was the same across all mediums. XD followed best practices for information transparency, designed documents for each medium with different subsets of engaging elements, and adapted them for the mediums (ie, additional ellipses between comic text) for the purposes of the study, consulting coauthors during design. (a) The video (Multimedia Appendix 2) was created to test the use of color, audio, and animations and illustrate the text using free resources on Biteable website (eg, “a doctor will call you” conveyed as an animation of a waving physician). The audio was provided by AS (a native German speaker) out of convenience. (b) The infographic (Figure 2) was designed with a step-by-step format and color from a health template on Canva (Canva Pty Ltd), with icons describing the text (eg, scheduling an appointment had a calendar icon; Multimedia Appendix 3). (c) The comic (Figure 3) used a story element and color and was designed in Figma with input from all authors, and it used simple figures to expedite the creation of the comics. The drawings sought to describe the text as literally as possible (eg, “you will be contacted” depicts a ringing phone; Multimedia Appendix 4). (d) The newsletter (Figure 4) used open format and color and was created in Figma (Figma, Inc; a popular website for user interface or user experience design) based on an existing newsletter template’s structure. The newsletter was thought to be a more familiar medium with more graphics than text (eg, newsletters sent via email; Multimedia Appendix 5).

### Study Design

All interviews took place via a web conferencing system. No recordings were taken, and a summary transcription was written after each question and finalized after each interview. This method was chosen to respect participant anonymity and COVID-19 protocols.

The interviewer invited participants to imagine that they were contacted by a data intermediary to obtain their consent to share their name, email, and allergy information with a hospital that wanted to carry out a clinical trial for lactose allergies. A verbal explanation was given along with the full plain text version of the consent form, and participants could ask questions at any time.

To answer RQ1, participants were asked about their previous experience with consent forms and desires regarding consent.

To address RQ2, after participants were shown all mediums of consent, they were asked to rank 8 design elements of a consent form: the use of colors, audio, animated elements, readability of text (eg, if it is not too technical or complicated), story element (eg, using examples and people in the forms), structured sections, step-by-step elements (eg, having an order to the information with text or visuals), and an open format (eg, being able to skip around to sections) from the most to the least engaging with an option for “other.”

To answer RQ3, we showed them a subsection of the full consent form, “What happens if I agree?” in different mediums (ie, comic, infographic, plain text, newsletter, and video versions) in a random order per participant. Participants were asked to rank the different forms according to their preferences and clarify why.

### Data Analysis

The interviews were documented in German, and anonymized answers were translated into English via DeepL (DeepL SE) and proofread by AS to ensure the translations’ adherence to the original meaning and to collaboratively analyze them with XD, AR, and MB (all non-German). Translation verification continued throughout the qualitative coding process in various sessions from November 2021 to April 2022 with the multidisciplinary team. To code the interview, the software MAXQDA (VERBI GmbH) was used. The expertise of the coding team spanned data protection law, usable privacy, bioethics, bioinformatics, and legal design.

To code the interviews, we inductively and iteratively established a codebook over three 2-hour sessions (Multimedia Appendix 6). The codebook combines a bottom-up approach through analysis of the data (eg, the concept of trust stemming from participant answers) with a top-down approach derived from the criteria for good documents given by Waller [26] (Table 2) to answer RQ3 (c).

**Table 2:** Document quality criteria elaborated by Waller [26].

Criteria			Description
**Language**			
	Directness	Using direct language to make it clear who is acting	
	Plain words	The extent to which the vocabulary is easily understood	
	Grammar	Conformity with good standard English practices	
	Readability	Ease with which the reader can follow arguments	
**Design**			
	Legibility	Use of legible fonts and text layout	
	Graphic elements	Use of tables, bullet lists, graphs, charts, icons, etc	
	Structure	Quality of document organization for function	
	Impression	Attractiveness and approachability, overall appearance	
**Relationship**			
	Who from	Is it clear who is communicating?	
	Contact	Whether there are clear contacts or means of contact	
	Audience fit	Appropriateness to the knowledge and skills of users	
	Tone	Matching the style and language of the context	
**Content**			
	Relevance	How relevant is the content to the recipient?	
	Subject	If it is clear what the communication is about	
	Action	Clarity about what action is required of the user	
	Alignment	Compliance with the organization’s intended aims and values	

Participant consent expectations have been organized into archetypes depending on the salience of reported goals and relevant features. A matrix was created with the participant number, expected features, expected goals, and expected behaviors to help group similar profiles.

### Ethical Considerations

The study design has been authorized by the Research Ethics Committee of the University of Luxembourg (ERP 21-038 LeAds), and best practices were followed. We chose a summary transcription to enable easier anonymization of the interview. Once manually anonymized, transcripts were securely shared with the authors from the other organization. The interviewees were compensated €30 for their time.

## Results

### User Desires via Archetypes

**Figure 5:**
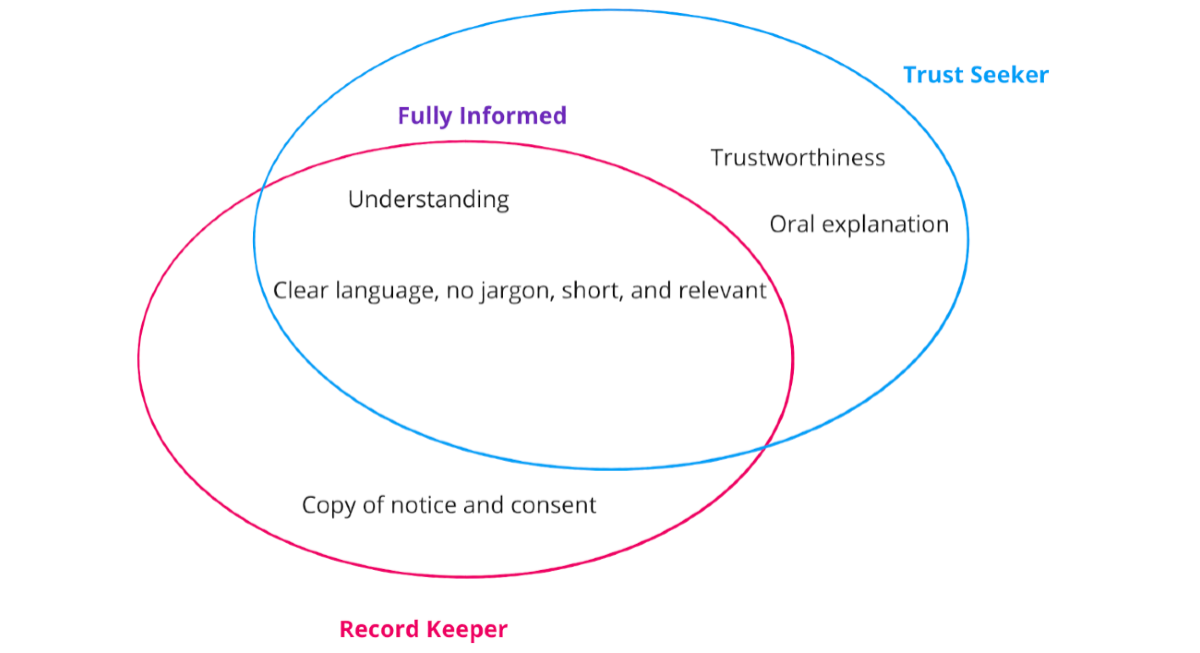
Venn diagram describing the core goals of different consent archetypes.

The interview findings from the questions, which explore participant expectations, desires, and needs, have been organized into 3 goal-oriented archetypes: the Fully Informed, the Record Keeper, and the Trust Seeker. Not all participants reported specific goals, while some participants reported multiple goals. Thus, the archetypes are based on grouping similar features (Figure 5).

The Fully Informed archetype wanted relevant and fitting information to understand what they were consenting to. This aligns with the most common goal explicitly reported by participants (14/24, 58%):

[A]s an affected person, I would like to see a few examples to get a better understanding of what may be done with my data.

P1

The information must also be appropriate for them as an audience:

A simple explanation that everyone understands would be my preference.

P12

The Record Keeper sought understanding while specifically wanting to remember what they had agreed to (3/24, 13%) or to have a copy for their records (4/24, 17%). For example, participant 13 had a clear idea of the elements they wanted to understand and retain a clear memory of:

It needs to be clear to me what the consent is for, who it is from, and exactly what data is being processed for what purpose.

P13

In addition, participant 4 stated the following:

It doesn’t matter to me if it is paper or digital. The main thing is that I receive a copy of the text to which I have consented.

P4

The Trust Seeker also sought understanding but was cautious toward the system or desired a trustworthy system (6/24, 25%):

I must have the impression that the data trustee is a reliable company or that there is an expertise that proves that I can trust this data trustee.

P3

[I would rather avoid] to invest time and read through stuff...[and be able to trust] since I’ve already given my data...that my data will just be handled well.

P7

When considered together, the archetypes lie on a spectrum where the Fully Informed archetype relies more on individual responsibility and capacity to make informed decisions, while the Trust Seeker also considers the context of organizational reputation and trust in making their decisions. In addition, the Record Keeper could be seen as an individual who wants to manage their consent decision over time, while those who do not want to review or revise their consent accept a one-time decision without records.

In addition to finding patterns based on common goals, some individuals stood out for their unique consent desires, including using more technical jargon (2/24, 8%). The use of jargon seemed to enable the process more time saving for some participants:

If I had to choose between short technical language and simple but longer language that is easy for everyone to understand, I would choose the short technical language.

P15

### Top Engaging Elements

**Figure 6:**
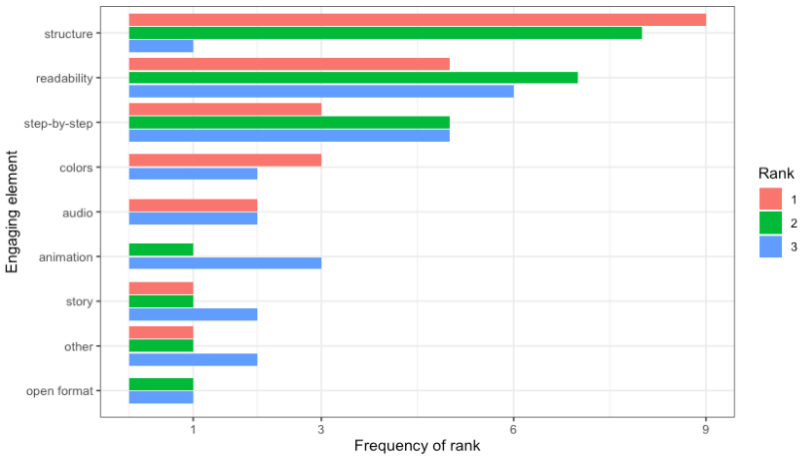
The frequency of each engaging element ranked from 1 to 3 by participants.

To better understand RQ2, about how different elements across mediums were perceived by participants, they were asked to rank the listed elements after experiencing all mediums. The most frequent element ranked first was structure, followed by readability, colors, step-by-step elements (tied with “colors”), audio, story, and others (also tied with “story”; Figure 6). The top element at rank 2 was also structure, and the top element at rank 3 was readability. When the option “others” was chosen, not all participants elaborated on what “other” element they referred to, but when they did, personal engagement (4/24, 17%) was most commonly cited. In ranks 2 and 3, structure, readability, and step-by-step were also frequently cited engaging elements.

### Preferred Mediums and Document Criteria

#### Overview

**Figure 7:**
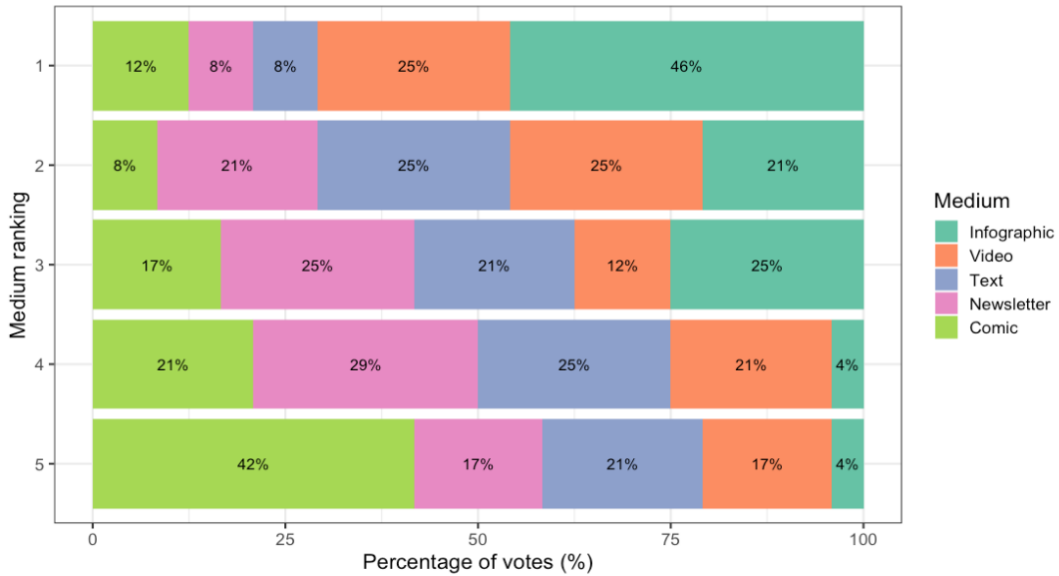
Participant ranking of mediums (where 1 corresponds to the first choice and 5 to the last choice) by percentage of votes.

To answer RQ3, we report the results about participants’ ranking for their preferred consent form after being shown each medium in Figure 7. The infographic was the overall winner and the comic the overall loser, while the video, text, and newsletter had varying trends (eg, the text was uniformly distributed across ranks 2 to 5, while the video was most often ranked 1, 2, or 4). Interestingly, no medium had consensus across the 24 participants.

In the following sections, each medium is discussed based on (1) the top 3 factors that influenced the ranking and (2) the top 3 positive or negative document criteria adapted from good document criteria by Waller [26] (Table 1). A participant could share multiple influencing factors or document criteria. We instead looked at the number of unique coded segments within their answer. Participants could share as few (though they were prompted to try to give at least 1) or as many factors as they desired. The coded segments for influencing factors, such as the element of time, could be positive (time-saving) or negative (time-wasting). This was to help us identify the categories that were most important to participants. Then, we contextualize the data and report whether important factors were positive or negative and their respective coded segments volume in the detailed section. Finally, Table 3 offers a summary of rank, top influencing factors, and document criteria per medium.

**Table 3:** Overview of the top 3 influencing factors and document criteria per medium with overall participant ranking.

			Medium								
			Infographic (rank 1)		Video (rank 2)		Text (rank 3)		Newsletter (rank 4)		Comic (rank 5)
**Influencing factors**											
	Positive element	Understanding, time, and interest		Understanding and effort		Understanding and time-saving		Prioritization and understanding		Understanding and interest	
	Negative element	N/A^a^		N/A		Boring		Association with advertisements		Inappropriate fit for the context	
	Mixed	N/A		Time-saving and time-wasting		N/A		N/A		N/A	
**Document criteria**											
	Positive element	Numbered lists, icons, bold headings, and graphic elements		Audio, step-by-step elements, and interplay of text and graphics		Structured layout		Bolded key text, sections, and open format for skimming		Text and graphics	
	Negative element	Extraneous or leading icons		N/A		Lacked highlighting		Advertisement impression		Tone and audience fit	

^a^N/A: not applicable.

#### Infographic Medium

The infographic was strongly preferred, with one-third of the participants (15/49, 31%) citing understanding as a positive influencing factor, while time and interest were in a close second. Elements such as numbered bullet points, bold headings, and icons were referenced:

With the bullets, you know right away what each is about in the text written underneath. In general, this is easy to grasp...

P3

The top 3 influencing factors were overall positive, in contrast to the other mediums.

Most positive document criteria concern design criteria such as step-by-step elements, icons, bold headings, bullet points, and color. There were much fewer negatively received elements, which were also related to the design criteria: the overuse of color and icons and the large size of the infographic. Participants had specific reactions to different icons, such as the hospital or medical professionals at the top and bottom that did not support any text or specific icons that might seem manipulative:

[W]ith the consent form, the “thumbs up” graphic makes it look like I’m being preempted from making a decision.

P14

#### Video Medium

Approximately one-third of the participants (14/44, 32%) reported that it influenced their understanding, followed by time and effort. Understanding was largely positive, partially due to the format that they’re “forced to watch it from beginning to end, so that you perceive the whole content” (P15).

On the other hand, time was slightly more positive than negative because while most participants felt that compared to reading, the video saved time, some felt it was inefficient compared to their reading speed, or they wanted to review material but felt rewinding would be time-wasting. Saving effort was wholly positive, with participants saying that it was more accessible, entertaining, or less attention draining while still being understanding. One minor interesting influencing factor unique to the video was a perceived feeling of trust from the audio, with 2 participants mentioning that a human voice engendered confidence in the process.

More than half of the positive feedback about the video mentioned the audio element, followed by the sequential nature and use of animation and images. Less than one-fourth of the participants (12/54, 22%) liked the content, which included the interplay between text and graphics and the story element:

What I like about the video is that...you see movements that show what you hear at the same time via audio.

P3

There were about half as many negative elements as positive ones, and most were due to the video pacing. Some wanted it faster, while others wanted it slower. Interestingly, 1 participant noted the following:

I have the feeling that with a video like this, people are rather uncritical of the content of the consent form. One is rather tempted to agree to something. If, for example, a button appeared after the video that allowed me to consent, I would probably consent.

P17

#### Text Medium

Approximately one-third of the participants (12/42, 29%) indicated that interest and understanding were most influenced by the text. Interest was a complex influencing factor that was slightly more negative. Those stating that it negatively influenced attention felt that the text medium was boring or lacked interest compared to other mediums. The participants who viewed it positively said that the text had a simple, clean layout allowing for quick skimming, and those who felt it was neutral felt like participant 21:

This is the format that I know and have simply accepted by now.

P21

The understanding was generally a positive influencing factor, with many saying that it was clear, concise, and short; however, some felt that it was difficult to skim or that the text was confusing or dry. Some participants also felt that it saved time by being short and concise.

The most cited positive document elements of the text were the use of clear sections, headlines, and bullet points. The positive elements were twice as common as the negative elements; the majority of both positive and negative elements also stemmed from design. Participants wanted more highlighting of key facts via colored, bold, italicized, or underlined words. Less than one-third of the participants (5/18, 28%) also cited the negative impression the document gave them:

[I]t is still a bit boring and trivial, so you might not read it properly if you get it as a letter home, for example.

P5

#### Newsletter Medium

More than a quarter of participants (6/22, 27%) mentioned prioritization as an influencing factor, less than a quarter (5/22, 23%) mentioned understanding, and 18% (4/22) mentioned interest. Prioritization and understanding were positive influencing factors, with participants saying that the bold words and ability to skip sections allowed them to roughly understand the contents because the bold text highlighted the important information in sentences. However, the interest factor was equally mixed, with the positive influence surrounding the bolded text and headers, while the negative influence was mainly attributed to the association with advertising spam. More than half of the participants (13/24, 54%) agreed with participant 1, who stated the following:

It looks like advertising, by the structure and the “headline,” which is repetitive.

P1

Although it had positive influencing factors, the negative interest likely had a large impact on the lower ranking of this form, as participant 5 said the following:

[I]t looks like advertising and I don’t like that.... I am rather annoyed by it. The bold as highlighting and the textual design I find good.

P5

The newsletter’s positive elements were largely regarding the design and use of structure, headings, bold text, sectioning, and the open format for skimming. The negative elements also similarly mentioned the design criteria because it looked like advertising based on prior experiences. The use of color was also disliked because the black header was too strong and off-putting.

#### Comic Medium

The main influencing factor was understanding, with one-third of the participants (7/24, 29%) mentioning it both positively and negatively. A slight majority (4/7, 57%) cited a positive influence on understandability. Interest was generally a positive influencing factor because the medium was novel. Less than one-fourth of coded segments showed that the comic had an overall negative influence on skimming, as the narrative-driven step-by-step format made it difficult to prioritize, reread for specific elements, or gain a quick overview. In addition, many participants disliked the comic medium as a whole, even if they could find some helpful design elements:

I found the comic a bit inappropriate for the topic...the message is better visualized by the little pictures, which may be better remembered but I don’t like it.

P20

Almost half of the positive feedback for the comic stemmed from the support of text with graphics, narrative elements, and illustrations. A third felt that the tone and audience fit suited them. However, negative impressions were almost double the positive ones because audience fit and tone were unsatisfactory for more than half of the participants (14/24, 58%):

I’m out of the age where I still like comics.... I don’t feel like I’m being taken seriously as a customer with a consent form like this.

P16

Participants suggested that children or older adults might be a better audience fit. Other negative feedback arose from the impression and graphic elements concerning the execution of illustrations, legibility, and lack of structure.

## Discussion

### Principal Findings

Qualitative analysis of participant desires for health consent revealed 3 archetypes: the Fully Informed, the Record Keeper, and the Trust Seeker. All participants wanted a high level of understanding before the consent decision, with some valuing additional elements such as obtaining copies of their decisions for their records and the trustworthiness of institutions like hospitals. The participants greatly stressed the need for short, concise, and direct consent forms that should not be longer than a page. Our results support the results of other authors, who have found that participants want to skim consent forms because consent documents are all the same, they want to save time, or they trust the ethical review of the related study [40]. In other words, individuals often engage in a form of strategic reading [26] instead of relying on attentive reading. This is why consent should contain elements that allow the visual prioritization of certain content over others, like headings, bullet points, and highlights (ie, “surface-level cues”) [26] that allow individuals to skim the document effectively and discern the most important information at first sight.

On the basis of the ranking of engaging elements, the participants preferred step-by-step documents (eg, linearly numbered lists with clear headings) instead of open or story-based formats. Structure, readability, and step-by-step elements were the top 3 engaging elements and could be easily integrated into most mediums. While our study only designed the infographic using 4 of the top engaging elements (ie, structure, readability, color, and step-by-step element), other mediums like text could also use color and step-by-step elements instead of the open-format element. However, the tone and audience fit of mediums greatly influenced participant rankings, even if some mediums enhanced understanding or visual interest (eg, comics and newsletters). The negative connotations of the newsletter with marketing and comics with childishness contributed to their low rankings, while text was seen as routine and acceptable, if boring. Instead of prioritizing one medium over another, there could be a greater focus on including the most important engaging elements within mediums (eg, adding step-by-step elements in all possible mediums).

### Implications for Practice

First, the creation of data-informed archetypes can be used for better understanding, and therefore accommodating, the diverse needs of a population. To leverage information describing the use of one’s own personal data as a self-determination instrument, individuals can receive contextualized information and concrete examples that are relevant to their specific needs (eg, the Fully Informed and Trust Seeker archetypes), rather than one-size-fits-all terms. Archetypes also support general audience tailoring for different goals. Different approaches to consent notices may reflect strategies to cope with the 2-fold reality stemming from the fact that the risks of consent decisions are individual, while the data sharing and processing are networked across the individual, responsible institutions, and beyond [41]. For example, the Fully Informed archetype may be more concerned with individual responsibility and the personal data processing, while the Trust Seeker wants information about the organizations involved and their security and privacy measures. Using archetypes to base user profiles could also be a way to customize their experience in meaningful macrocategories without needing to customize every possibility for individual preferences. However, more research is needed to balance the actual benefit of tailoring information to different learning styles [42] against the increased costs of its creation and implementation.

Second, different mediums can be targeted based on needed affordances (Table 3) and layered to reinforce the understanding of complex information, for example, through a multifold presentation of the same content through text, video, and infographics. Official guidance about transparency requirements’ implementation [15] portrays layering techniques as an appropriate means to achieve the requirement of full disclosure while allowing for prioritization and brevity. For example, summaries containing an overview of the main clauses can accompany the more comprehensive version and can be more easily browsed while consenting, with short videos and privacy icons constituting the first layer of a written notice [20]. Distributing information on separate mediums can additionally contribute to presenting the relevant information at an appropriate time. For instance, the first layer with essential information can be displayed at the moment of the consent decision, while detailed information can always remain accessible on request [43]. However, as more guidelines for multimedia consent design arise [14,20], testing and co-designing with the intended audience is key; otherwise, a medium with a negative audience fit for certain contexts may be less effective than plain text consent (eg, the unsuitable comic medium for German adults). This can be important to test for among the intended audience, especially as comics have been a case study for cultural stigmas [44]. While they have been suitable for Indigenous populations [15], some researchers are pushing for more serious comics (similar to serious games for education) [45] and the comic co-design process itself as a research practice [46].

In terms of implementation, layering has been integrated with dynamic consent platforms. Dynamic consent was built to leverage the benefits of digital communication for health research by using digital platforms to connect people and researchers and allow participants to view, update, and change their data sharing permissions dynamically. Australia’s CTRL [36], a dynamic consent platform based on open-source code, incorporates multimedia (video, illustrated text, and infographics); personalization options; and informational layering techniques. Building upon this, the layering could incorporate archetypes of general profiles to be tailored for different goals. Users of different ages may prefer different mediums, such as comics for younger audiences and videos for older audiences; similarly, users with domain expertise could choose content explained with jargon.

Finally, although we did not explicitly ask about undue influence of design elements on consent decisions and trust implications, participants clearly connected the 2, and more research is needed to better understand the deep connection. The infographic had a few complaints about specific graphics, with participant 14 saying that showing a “thumbs-up” icon was perceived as a manipulative way to preempt one from making an informed decision. Similarly, participant 17 stated that participants might believe anything shown in a video and be inclined to give consent. While guidance on ethical nudging design [47-49], as well as research on dark patterns that are to be avoided [7,8], can help shed light on such thorny issues–the issue should be more deeply studied. Considering how often human beings take decisions that are not completely rational [50,51], adding elements such as icons, color, or audio may increase the potential for manipulation of choice.

### Limitations

Although we strove to obtain balanced age, education, and sex representation in our sample, they cannot be fully representative of the population. More research should be done on populations other than German adults with a larger sample size. It should also be replicated in the specific consent context of interest, as our study focused on consent to share personal data for further contact; replicating the study for clinical trial consent is important, as it may offer new archetypes, rankings, and contextual concerns. More research should also be done to study how to refine and apply archetypes in practice, as it can be insufficient or biased without continued user, expert, or patient input. Our methods only concern self-reported opinions, so there may be a discrepancy between reported preferences and observed behaviors. The study materials may have influenced rankings and preferences, as they were generated by XD, who is not a professional designer. Therefore, certain choices (eg, the simple comic style) could have influenced participants’ attitudes, making it difficult to determine the exact stimulus based on self-reported answers. While out of scope of this work, future studies can research how specific design elements or stereotypes impact rankings, for example, showing a comic with stick figures or realistic figures to German adults to better understand how to design the specific element. Before implementing consent mediums in line with applicable constraints, relevant expertise should be included in the design and evaluation of each medium.

### Conclusions

To better understand the diversity of participant preferences, opinions, and emotions for IC in a health care scenario and the relevance of specific document criteria for engagement with various mediums (ie, infographic, video, text, newsletter, and comic), this study interviewed 24 individuals. The results not only have informed the generation of archetypes based on desired document features and goals but can also help create standardized consent documents that use layering to help address varying needs identified via archetypes. We also proposed recommendations for designing multimedia consent forms with a structure that promotes prioritization, such as headers, bullet points, and bold type within a contextually appropriate medium, such as an infographic or a video, so that the forms are seen by our participants as more attention-grabbing and serious than comics. It would be important to replicate this study setting in other countries, and the results could lead to contextually designed consents that align with the GDPR and other EU regulations. The findings reported here are meant to encourage further research to determine how to better involve individuals in designing useful, engaging consent forms to facilitate informed decisions concerning data sharing.

## Appendix

Multimedia Appendix 1 Interview guidelines in English.

Multimedia Appendix 2 Full video used in the study.

Multimedia Appendix 3 English version of the infographic used.

Multimedia Appendix 4 English translation of the comic shown in the study.

Multimedia Appendix 5 English version of the newsletter shown in the study.

Multimedia Appendix 6 Full codebook used in analysis.

## Abbreviations

EU: European Union
GDPR: General Data Protection Regulation
IC: informed consent
RQ: research question
