# Supplementary material for: Comparing Attitudes Toward Different Consent Mediums: Semistructured Qualitative Study
Source: JMIR Hum Factors. 2024 Apr 30;11:e53113. doi: 10.2196/53113 (PMC11094594; doi:10.2196/53113)
Supplement: Multimedia Appendix 1 [file humanfactors_v11i1e53113_app1.docx]

**Interview guideline (Translation trough deepl.com – interviews will be held in German)**

| **Data protection information**  This data protection information applies to the interview conducted by the Fraunhofer-Gesellschaft zur Förderung der angewandten Forschung e.V., Hansastraße 27 c, 80686 München (Munich, Germany) for its Fraunhofer SIT and for its Fraunhofer IGD, Tel.:06151-869-100, E-Mail: info@sit.fraunhofer.de.  The data protection officer of Fraunhofer can be contacted at the address Fraunhofer-Gesellschaft zur Förderung der angewandten Forschung e.V., z. Hd. Datenschutzbeauftragter, Hansastraße 27 c, 80686 München, Germany, or at datenschutz@zv.fraunhofer.de. You can contact our data protection officer directly at any time.  The records of your interview are processed for research purposes. The written records are processed on the basis of our legitimate interest in carrying out research in the field of IT-Security and privacy (Art. 6 para. 1 lit. f GDPR). All possible other records are only being carried out and processed on the basis of your explicit consent (Art. 6 para. 1 lit. a GDPR).  Your personal data will be stored as long as we are legally obliged to do so, especially in regard to legal storage obligations arriving from tax laws. Anonymized records can be stored for an indefinite period of time and can be shared with other research institutions as well as published in scientific journals and conferences.  You have a right to information, correction, erasure, restriction of processing and data transferability with regard to personal data concerning you. Furthermore, you have the right to object to the processing that is based on our legitimate interest. If you gave us consent to record your interview – or parts of your interview – you can revoke your consent at any time for the future. If you wish to exercise your rights, please send an E-Mail to datenschutzkoordination@zv.fraunhofer.de.  You also have the right to complain to a data protection supervisory authority about our processing of your personal data. |
| --- |

Thank you for taking the time to answer some questions for me today about consent to the processing of personal data. Personal data are those data that relate to you personally, such as your name, date of birth, and disease diagnoses.

Please let me start the interview by saying who I am, who I am carrying out the interview for and what the purpose of the interview is:

- Introduction Annika (personally)
- Introduction Fraunhofer SIT
- Introduction University of Luxemburg
- Introduction of research questions (roughly summarized, as for the target group).

Your participation in this interview is voluntary and you can stop the interview at any time. If you stop the interview at any point, the entire information we gained during your interview will be erased and not used further.

| The following information will be used exclusively for the evaluation and documentation of the interview by Fraunhofer SIT and the University of Luxembourg. This data will explicitly not be published in scientific articles.  Date of the interview:  For internal evaluation, if interviewee wants to provide this data:  Name of interviewee:  One of our goals is to find out whether or not factors like income influence the way a person judges a consent form. Therefore, it would help us if you would let me know your annual income. When we publish the interview results, none of the information you give us today – including the information about your income – will be linkable to you personally.  Annual income of interviewee:   1. My income is under 20.000€ 2. My income is over 20.000€ and under 40.000€ 3. My income is over 40.000€ and under 60.000€ 4. My income is over 60.000€ and under 80.000€ 5. My income is over 80.000€ and under 100.000€ 6. My income is over 100.000€ |
| --- |

The following interview will be recorded in writing. The interview evaluation is published in scientific articles, some of which are also available worldwide via the Internet, in German or English. The publication will take place without mentioning your name.

It is no problem if you cannot or do not want to answer one or more of the questions I asked - if you cannot or do not want to give an exact answer to a question, I may ask you for a (rough) estimate.

At some points in the interview, you will be asked if you agree to have your screen recorded so that we can see how you moved your mouse even after the interview is over. If you give your consent to screen recording, we will ask you to turn off the camera that is recording you yourself before the recording begins.

**Question 1*^[[1]](#footnote-1)^**

Which of the following three answer alternatives applies to you?

a) My gender is:

b) I prefer not to reveal my gender for this interview.

c) I am between 18-30 years old.

d) I am between 31-55 years old.

e) I am between 56-90 years old.

f) The highest qualification I have achieved so far is a school-leaving certificate or vocational training at school or in a company.

g) My highest degree to date is a degree from a college or university.

h) Not three of the above answer alternatives apply to me.

*(there is a presentation of the data processing context*

- *Introducing the three actors of the data processing context:*
  - *Introduction of a data trustee*
  - *Introduction to hospitals (and clinical trials)*
  - *Introduction of the data subject (= the interviewee)*
- *Imagine you are allergic to lactose and this allergy causes you severe physical symptoms, especially nausea and strong headaches. A data trustee has your personal data (which was donated for research purposes from the clinic that treats your allergies), specifically your name, your address, your diagnosis and a list of your symptoms. A pharmaceutical company developed a new medicine against lactose allergy which is supposed to especially help patients that suffer from nausea accompanied by strong headaches. The hospital that leads the clinical trial asks the data trustee if he has data of people with lactose allergies and symptoms of nausea and strong headaches. The data trustee contacts you to ask your consent to hand out the data to the hospital, so that the hospital can contact you and invite you to participate in the clinical trial.* *When it comes to processing your personal data, a consent means any freely given, specific, informed indication of your wishes in regard to the processing your personal data)*

**Question 2***

Do you have any questions of understanding about the context in which personal data are processed, e.g. how they are stored by a data trustee and handed out to the hospital?

a) Yes, namely:

b) No.

**Question 3**

What do you know about consent to the processing of your personal data in general?

**Question 4**

What are your experiences with a website or hospital requesting your consent? Which emotions did you have while engaging with the consent (showing the emotion wheel in a power point slide for guidance)?


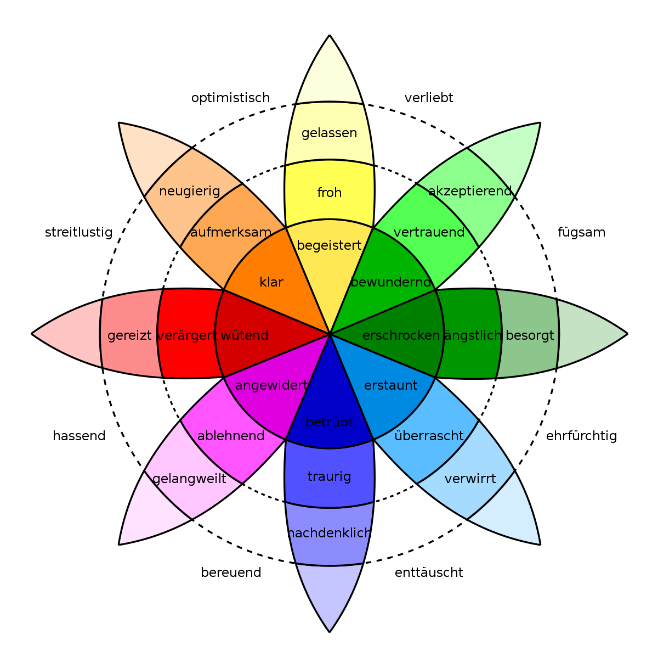


*Rad der Emotionen nach Robert Plutchik (Quelle: Wikipedia)*

**Question 5**

How long do you usually take time to engage with a consent form before you give or refuse your consent it?

a) I usually just give or refuse my consent, without engaging at all.

b) Less than a minute.

c) 1-5 minutes.

d) I take as long as it necessary to read trough and understand everything.

**Question 6**

What would be your expectations or preferences of consent in the context of the data processing context presented?

**Question 7 (if not already answered under 6).**

What would be your expectations of consent in relation to the text?

**Question 8 (if not already answered under 6)***

What would be your expectations of consent in terms of form (e.g. paper based, digital text, audio file)?

a) Digital vs. physical.

b) Audio vs written vs video.

**Question 9 (if not already answered under 6)**

What would be your expectations of consent in terms of engaging while receiving information?

**Question 10 (if not already answered under 6).**

What would be your expectations of consent in terms of being given relevant information transparently and giving consent?

*(There will be a presentation of the consent text (1) in plain text form).*

**Question 11***

Do you have any comprehension questions about the consent text?

a) Yes, namely:

b) No.

*--start recording if applicable--.*

*(there is a presentation of the consent text again (1) as text form formatted as newsletter as well as (2) as an infographic (3) in form of a comic and (4) in form of a video. During this part of the interview - i.e. the presentation of the four different consent texts - the interviewees are asked if they can show where they are looking with their mouse on the screen. Before beginning, interviewees are asked if they agree to this part of the interview being recorded)*

*--stop recording if applicable--.*

**Question 12**

Please rank the four different ways of consent form information in the order you prefer, from most favorite to least favorite.

**Question 13**

(Re #1 in the order).

What do you like about the consent form and why? To what extent does it meet the expectations you have for consent in terms of text and form? How did you experience the consent – which emotions did you have while experiencing the consent (showing the emotion wheel in a power point slide for guidance), which elements were particularly enjoyable, which elements helped you actively engage with the consent? Are there elements of the consent form that you don't like and why?

**Question 14**

(Re #2 in the order)

What do you like about the consent form and why? To what extent does it meet the expectations you have of consent in terms of text and form? How did you experience the consent which emotions did you have while experiencing the consent (showing the emotion wheel in a power point slide for guidance), which elements were particularly enjoyable, which elements helped you actively engage with the consent? Are there elements of the consent form that you don't like and why?

**Question 15**

(Re #3 in the order)

What do you like about the consent form and why? To what extent does it meet the expectations you have of consent in terms of text and form? How did you experience the consent which emotions did you have while experiencing the consent (showing the emotion wheel in a power point slide for guidance), which elements were particularly enjoyable, which elements helped you actively engage with the consent? Are there elements of the consent form that you don't like and why?

**Question 16**

**(**Re #4 in the order**)**

What do you like about the consent form and why? To what extent does it meet the expectations you have of consent in terms of text and form? How did you experience the consent which emotions did you have while experiencing the consent (showing the emotion wheel in a power point slide for guidance), which elements were particularly enjoyable, which elements helped you actively engage with the consent? Are there elements of the consent form that you don't like and why?

**Question 17**

You saw and commented on four different consent forms during this interview. Is there anything you did not see, but would want to see in a consent form?

**Question 18**

Please rank the following elements of a consent form in the order that you feel they add to your engagement with the consent form (from best engagement to the least engagement):

1) Use of colors

2) Audio elements

3) Animated/Moving elements

4) Readability of text (e.g. if it is not too technical or complicated)

5) Story element (e.g. using examples and people in the forms)

6) Structured sections/headers

7) Step-by-Step elements (e.g. having an order to the information with text or visuals)

8) Open format (e.g. being able to skip around to sections)

9) Other

**Question 19**

Would the elements of a consent form that you saw today make you spend more attention or less attention, or find more interesting, or increase understanding, or increase engagement with a consent form before you sign it? If so: which ones and why?

**Question 20**

How would you want to manage (e.g. withdraw) consent you have already given? E.g., by logging into a data management platform, by sending an email/letter, clicking a button, signing a piece of paper, ...? Why would you prefer this method of giving consent?

*(Explanations follow on the fact that consent to personal data processing, once given, can be revoked and what role a data management platform could play in this).*

**Question 21**

In what situations/circumstances would you want to revoke consent that has already been given? What considerations play a role for you in this regard?

**Question 22**

How would you want to revoke consent you have already given? E.g., by logging into a data management platform, by sending an email/letter, clicking a button, signing a piece of paper, ...? Why would you prefer this method of withdrawing consent?

Thank you for answering my questions!

Do you have any further questions for me or suggestions regarding my survey?

1. For questions marked with an asterisk (*), the alternative answers were given. For all other questions, the answer alternatives have merely been designed in order to be able to document the interviews according to a uniform scheme. [↑](#footnote-ref-1)
