# Supplementary material for: Comparing Attitudes Toward Different Consent Mediums: Semistructured Qualitative Study
Source: JMIR Hum Factors. 2024 Apr 30;11:e53113. doi: 10.2196/53113 (PMC11094594; doi:10.2196/53113)

WHAT HAPPENS IF I AGREE?

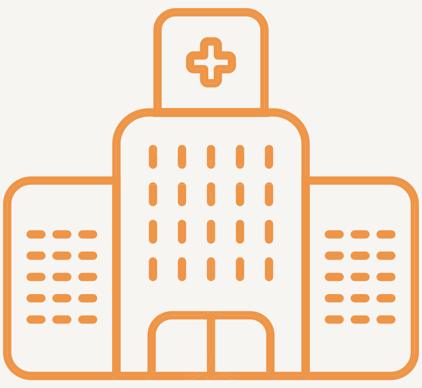

If you allow the trustee to share your contact with the hospital, the following will happen:

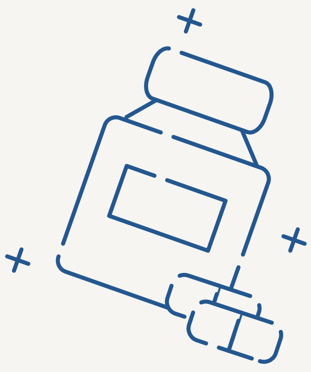

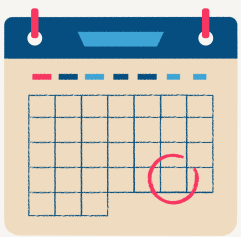

1 CONTACT

The hospital will contact you after receiving your information.

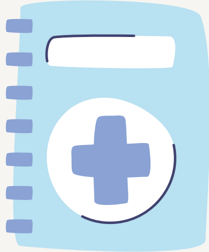

2 MEDICAL RECORDS

Usually, the hospital will ask you to bring all your medical records with you.

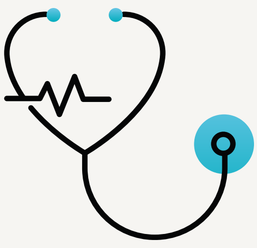

3 PHYSICAL EXAMINATION

Then you will be examined.

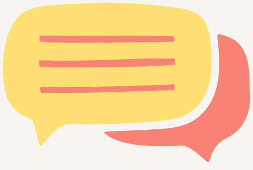

4 EXPLANATION

You will join an educational discussion about the trial.

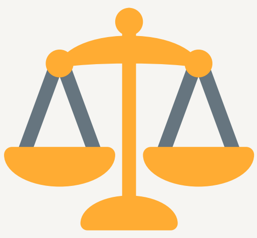

5 DECISION

Then you can decide to join the clinical trial or not.

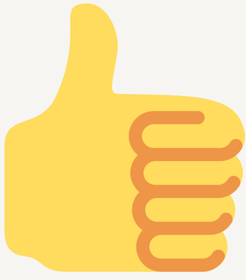

6 AGREEMENT

If you wish to proceed, you must consent to data processing by the clinical trial for research and monitoring your health.

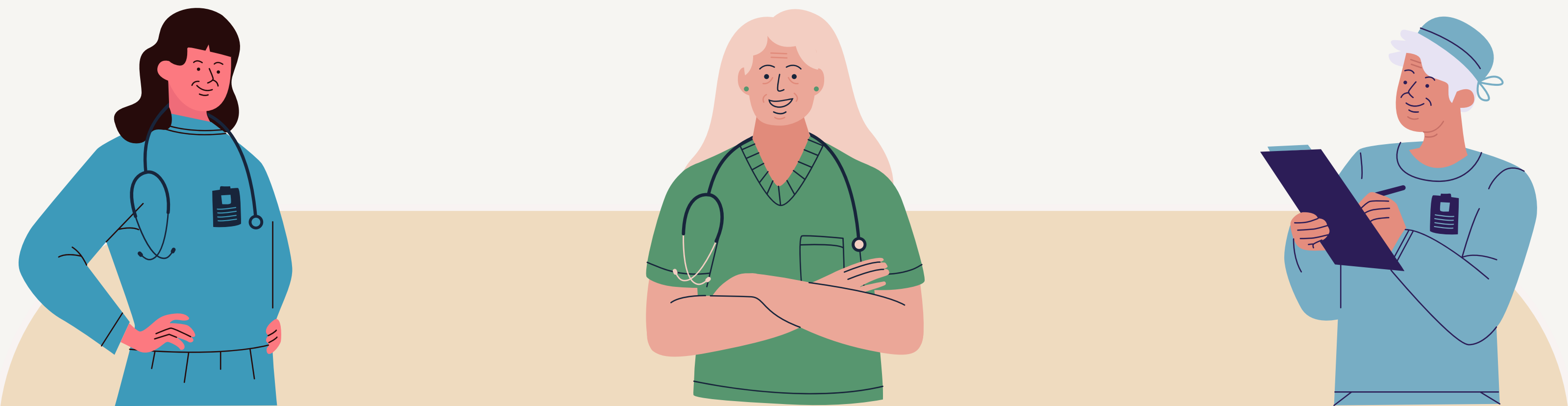

Supplement: Multimedia Appendix 3 [file humanfactors_v11i1e53113_app3.pdf]
