# Supplementary figures and images for: Comparing Attitudes Toward Different Consent Mediums: Semistructured Qualitative Study
Source: JMIR Hum Factors. 2024 Apr 30;11:e53113. doi: 10.2196/53113 (PMC11094594; doi:10.2196/53113)

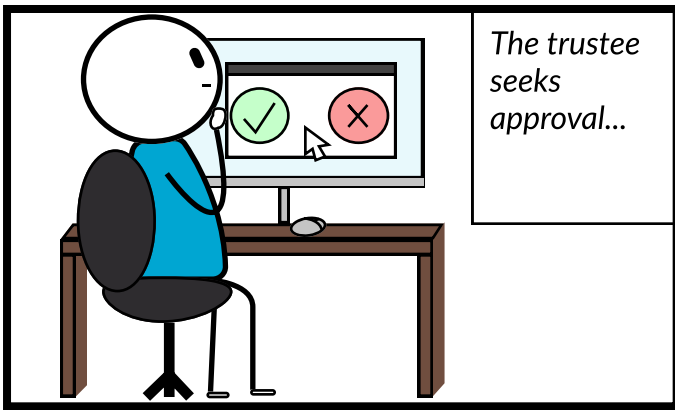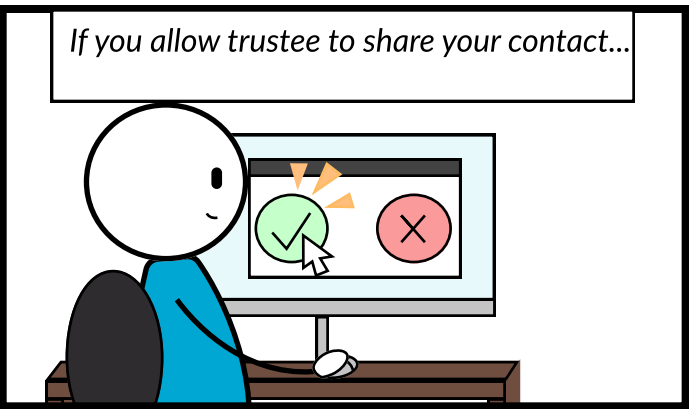

# What happens if I agree?

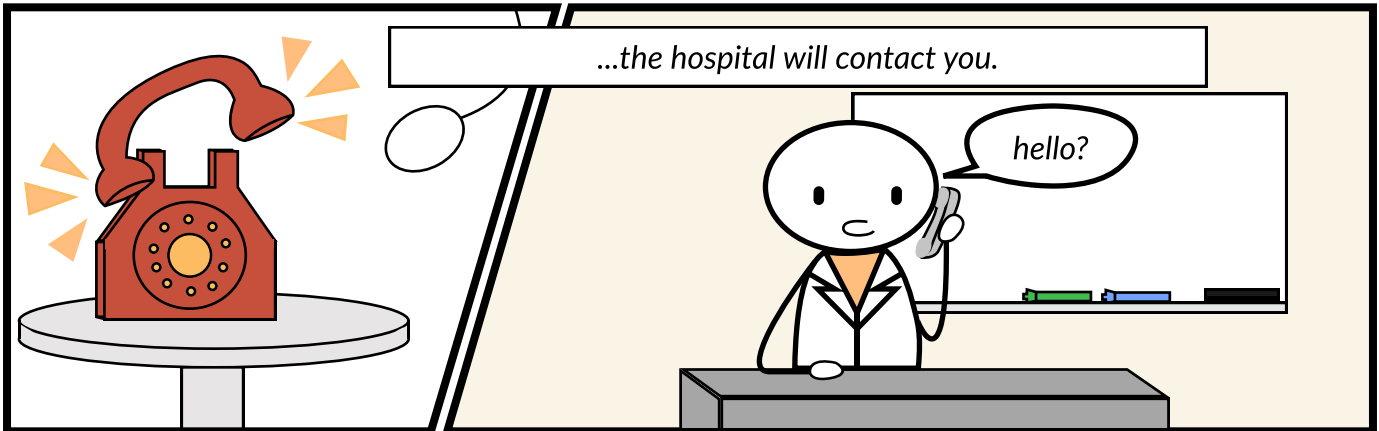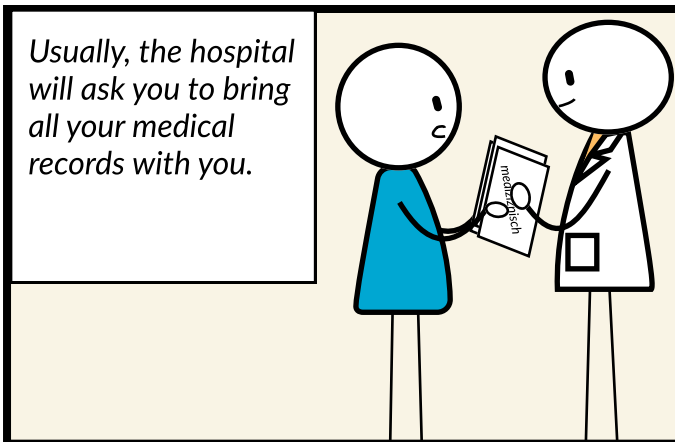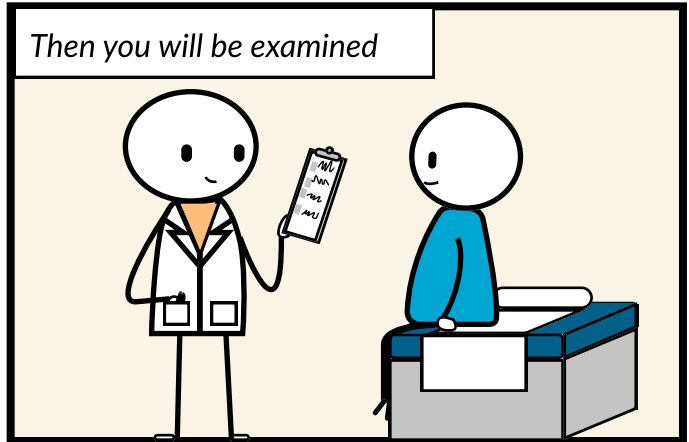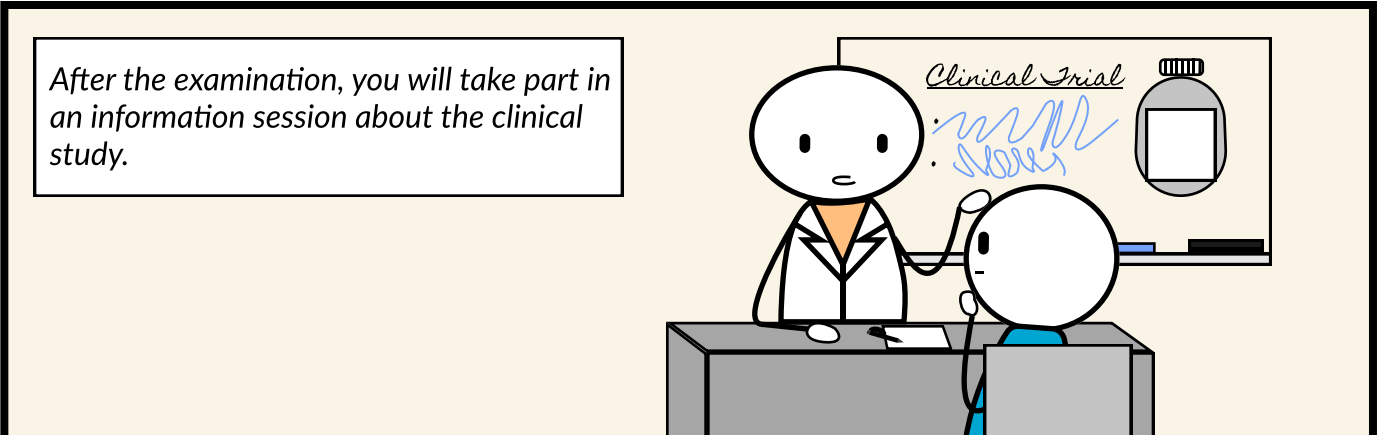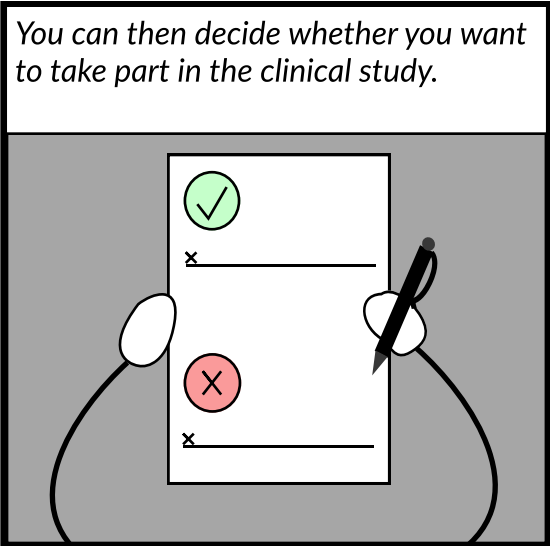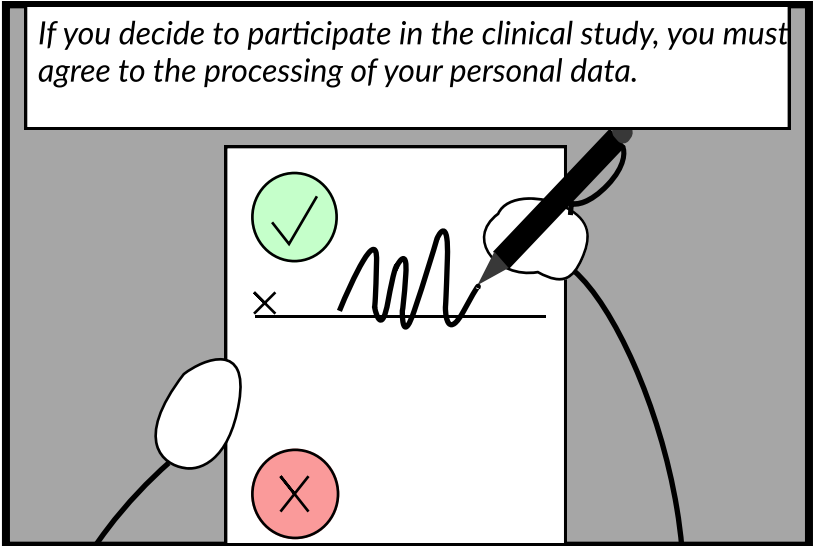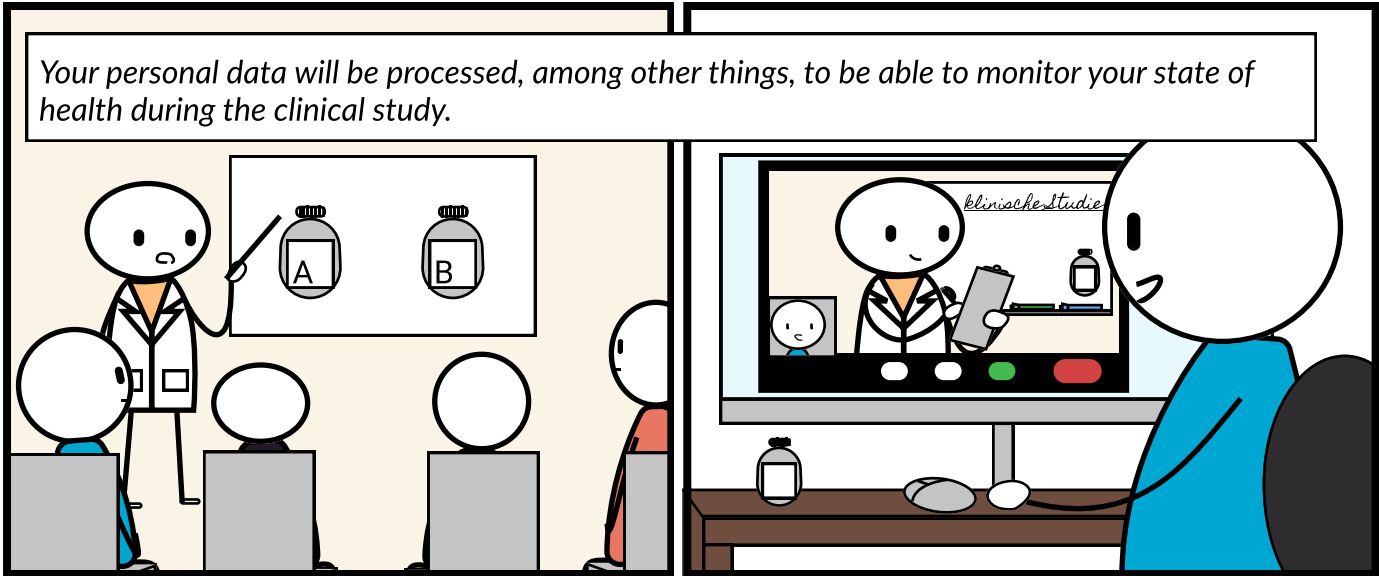

Supplement: Multimedia Appendix 4 [file humanfactors_v11i1e53113_app4.pdf]
