# Supplementary material for: Comparing Attitudes Toward Different Consent Mediums: Semistructured Qualitative Study
Source: JMIR Hum Factors. 2024 Apr 30;11:e53113. doi: 10.2196/53113 (PMC11094594; doi:10.2196/53113)
Supplement: Multimedia Appendix 5 [file humanfactors_v11i1e53113_app5.pdf]

Dear Person, you are invited to participate!

Dear Person

We are pleased to inform you that you can participate in a clinical trial that may be relevant to you, made possible by the secure transfer of your data through the Smart Cities Initiative of Test-Land.

What happens if I agree?

- The hospital will **contact** you.
- Usually, the hospital will ask you to bring all your **medical records**. Then you will be **examined**.
- After the exam, you will participate in an **educational interview** about the clinical trial. After that, you can decide if you want to participate in the clinical trial.
- If you decide to participate in the clinical trial, you must **consent to the processing of your personal data**. Your personal data will be processed, among other things, in order to monitor your health during the clinical trial.
- - We are XYZ Company, Test Street 5, 12345 Test City (Test Country).

These illustrations and texts are the result of a collaboration between the University of Luxembourg (X. Doan, A. Rossi, M. Botes, G. Lenzini) and Fraunhofer SIT (A. Selzer).
