# Supplementary material for: Comparing Attitudes Toward Different Consent Mediums: Semistructured Qualitative Study
Source: JMIR Hum Factors. 2024 Apr 30;11:e53113. doi: 10.2196/53113 (PMC11094594; doi:10.2196/53113)
Supplement: Multimedia Appendix 6 [file humanfactors_v11i1e53113_app6.docx]

Code System

| **Code System** | **Memo** | **Frequency** |
| --- | --- | --- |
| Code System |  | 1473 |
| Prior Knowledge of Data Processing | From: Question 3  What do you know about consent to the processing of your personal data in general?  Related to the type of information they share, e.g. data processing rights, GDPR rules, etc. NOT how much knowledge | 24 |
| Data Processing Regulations |  | 0 |
| Incorrect Knowledge of GDPR/Data Protection |  | 0 |
| GDPR Rights |  | 0 |
| GDPR exists |  | 0 |
| No Knowledge |  | 4 |
| Some Knowledge |  | 16 |
| Lots of Knowledge |  | 4 |
| Other |  | 0 |
| Prior Experience with Consent | From: Question 4  What are your experiences with a website or hospital requesting your consent? Which emotions did you have while engaging with the consent (showing the emotion wheel in a power point slide for guidance)? More about the in the moment experience. | 38 |
| Recruiting |  | 1 |
| Financial Consent |  | 4 |
| Healthcare Consent |  | 14 |
| Cookie Banners |  | 11 |
| None |  | 0 |
| General Experience | Go into emotios later if interesting | 8 |
| Time Spent On Consent |  | 70 |
| Context |  | 17 |
| Other Context |  | 4 |
| Type of Data Being Processed |  | 1 |
| Written/Physical/Doctor |  | 5 |
| Digital/Cookie |  | 7 |
| Reasoning |  | 33 |
| Trust |  | 6 |
| As much time to skim |  | 5 |
| As long as a necessary to understand |  | 10 |
| As little as possible to sign |  | 8 |
| Other |  | 4 |
| 0 sec |  | 2 |
| 0-30 sec |  | 5 |
| 30 sec - 1 min |  | 2 |
| 1+ min |  | 11 |
| Other |  | 0 |
| Expectations for Consent |  | 148 |
| Negative Emotions |  | 2 |
| Positive Emotions |  | 1 |
| Delivery Method |  | 0 |
| Digital |  | 17 |
| Physical |  | 14 |
| In Person/Oral |  | 8 |
| Content Criteria |  | 0 |
| Alignment |  | 0 |
| Action | what is actionable? What can you do or not do with the information given? | 2 |
| Subject |  | 0 |
| Relevance | Completeness in terms of what a participant wanted to know | 10 |
| Relationship Critera |  | 0 |
| Tone |  | 5 |
| Audience Fit |  | 12 |
| Contact |  | 1 |
| Who From |  | 0 |
| Design Criteria |  | 0 |
| Impression |  | 7 |
| Structure |  | 9 |
| Graphic Elements | Bold text, bullet points, graphic visual aids, etc | 19 |
| Legibility | More about font choice, font size, and margins | 1 |
| Language Criteria |  | 0 |
| Readability |  | 5 |
| Grammar and Punctuation |  | 1 |
| Plain Words |  | 8 |
| Directness/Conciseness | using brief/direct language to make it clear who's doing what | 21 |
| Other |  | 5 |
| Enables |  | 262 |
| Overview/Summary |  | 20 |
| Retention/Memorization |  | 9 |
| Identifying with a Character |  | 4 |
| Effort |  | 16 |
| Interest/Attention |  | 37 |
| Proof | Of understanding | 0 |
| Prioritization/Skimming |  | 41 |
| Time |  | 50 |
| Feelings of Trust |  | 13 |
| Consent Management |  | 8 |
| Interactivity |  | 2 |
| Understanding/Clarity |  | 62 |
| Experience During Study |  | 482 |
| Video |  | 101 |
| Video-Positive |  | 0 |
| Video-Positive-Video Pacing | Has to follow the video to understand information at a given pacing | 3 |
| Video-Positive-Content Criteria |  | 0 |
| Relevance |  | 1 |
| Story/Narrative Element |  | 2 |
| Interplay of Text and Graphics | add audio | 9 |
| Video-Positive-Language Criteria |  | 0 |
| Readability |  | 0 |
| Directness/Conciseness |  | 0 |
| Plain Words |  | 0 |
| Video-Positive-Design Criteria |  | 1 |
| Impression |  | 2 |
| Legibility |  | 0 |
| Graphic Elements |  | 6 |
| Use of Color |  | 1 |
| Animated/Moving Element |  | 2 |
| Audio Element |  | 13 |
| Structure |  | 4 |
| Step-by-Step Elements |  | 4 |
| Structured Layout |  | 0 |
| Open Format |  | 0 |
| Video-Positive-Relationship Criteria |  | 5 |
| Audience Fit |  | 1 |
| Tone |  | 0 |
| Unconventional Consent Process |  | 4 |
| Video-Positive-Other |  | 5 |
| Video-Negative |  | 0 |
| Video-Negative-Relationship Criteria |  | 5 |
| Audience Fit |  | 1 |
| Tone |  | 0 |
| Unconventional Consent Process |  | 4 |
| Video-Negative-Content Criteria |  | 2 |
| Story/Narrative Element |  | 0 |
| Subject |  | 0 |
| Interplay of Text and Graphics | + other elements (e.g. audio) | 2 |
| Video-Negative-Language Criteria |  | 0 |
| Directness/Conciseness |  | 1 |
| Plain Words |  | 1 |
| Readability |  | 1 |
| Video-Negative-Design Criteria |  | 0 |
| Impression |  | 0 |
| Legibility |  | 0 |
| Graphic Element |  | 1 |
| Use of Color |  | 0 |
| Audio Element |  | 2 |
| Animated/Moving Element |  | 0 |
| Structure |  | 0 |
| Structured Layout |  | 0 |
| Step-by-Step Element |  | 1 |
| Open Format |  | 0 |
| Video Pacing |  | 10 |
| Other |  | 1 |
| Personal Preference/Experience |  | 6 |
| Does Not Meet Expectations |  | 5 |
| Meets Expectations |  | 11 |
| Desires |  | 0 |
| Simple Narrative |  | 0 |
| Physical Consent |  | 0 |
| Digital Consent |  | 0 |
| No Abstractions |  | 0 |
| Other |  | 0 |
| Plain Consent Text |  | 79 |
| Plain Consent Text-Positive |  | 27 |
| Plain Consent Text-Positive-Content Criteria |  | 1 |
| Subject |  | 1 |
| Story/Narrative Element |  | 0 |
| Interplay of Text and Graphics |  | 0 |
| Plain Consent Text-Positive-Language Criteria |  | 3 |
| Readability |  | 1 |
| Directness/Conciseness |  | 2 |
| Plain Words |  | 0 |
| Plain Consent Text-Positive-Design Criteria |  | 23 |
| Impression |  | 3 |
| Legibility |  | 0 |
| Graphic Elements |  | 9 |
| Use of Color |  | 0 |
| Animated/Moving Element |  | 0 |
| Audio Element |  | 0 |
| Structure |  | 2 |
| Step-by-Step Elements |  | 0 |
| Structured Layout |  | 9 |
| Open Format |  | 0 |
| Relationship Criteria |  | 0 |
| Audience Fit |  | 0 |
| Tone |  | 0 |
| Unconventional Consent Process |  | 0 |
| Other |  | 0 |
| Plain Consent Text-Negative |  | 18 |
| Plain Consent Text-Negative-Content Criteria |  | 0 |
| Subject |  | 3 |
| Interplay of Text and Graphics |  | 0 |
| Plain Consent Text-Negative-Design Criteria |  | 0 |
| Impression |  | 5 |
| Graphic Element |  | 5 |
| Use of Color |  | 1 |
| Audio Element |  | 0 |
| Animated/Moving Element |  | 0 |
| Structure |  | 1 |
| Structured Layout |  | 2 |
| Step-by-Step Element |  | 0 |
| Open Format |  | 0 |
| Legibility |  | 0 |
| Relationship Criteria |  | 0 |
| Tone |  | 0 |
| Unconventional Consent Process |  | 0 |
| Audience Fit |  | 0 |
| Language Criteria |  | 0 |
| Readability |  | 0 |
| Other |  | 1 |
| Personal Preference/Experience |  | 16 |
| Does Not Meet Expectations |  | 4 |
| Meets Expectations |  | 14 |
| Desires |  | 0 |
| Physical Consent |  | 0 |
| Digital Consent |  | 0 |
| No Abstractions |  | 0 |
| Simple Narrative |  | 0 |
| Other |  | 0 |
| Newsletter |  | 96 |
| Newsletter-Positive |  | 45 |
| Newsletter-Positive-Content Criteria |  | 0 |
| Relevance |  | 6 |
| Story/Narrative Element |  | 0 |
| Interplay of Text and Graphics |  | 2 |
| Newsletter-PositiveDesign Criteria |  | 0 |
| Impression |  | 3 |
| Legibility |  | 0 |
| Graphic Elements |  | 3 |
| Use of Color |  | 4 |
| Animated/Moving Element |  | 0 |
| Audio Element |  | 0 |
| Structure |  | 25 |
| Step-by-Step Elements |  | 0 |
| Structured Layout |  | 18 |
| Open Format |  | 6 |
| Newsletter-Positive-Relationship Criteria |  | 0 |
| Who From? |  | 2 |
| Audience Fit |  | 0 |
| Tone |  | 0 |
| Unconventional Consent Process |  | 0 |
| Language Criteria |  | 0 |
| Readability |  | 0 |
| Directness/Conciseness |  | 0 |
| Plain Words |  | 0 |
| Other |  | 0 |
| Newsletter-Negative |  | 31 |
| Newsletter-Negative-Relationship Criteria |  | 1 |
| Who from? |  | 1 |
| Tone |  | 0 |
| Unconventional Consent Process |  | 0 |
| Audience Fit |  | 0 |
| Newsletter-Negative-Content Criteria |  | 4 |
| Relevance |  | 3 |
| Subject |  | 1 |
| Interplay of Text and Graphics |  | 0 |
| Newsletter-Negative-Language Criteria |  | 2 |
| Plain Words |  | 0 |
| Directness |  | 0 |
| Readability |  | 2 |
| Newsletter-Negative-Design Criteria |  | 0 |
| Impression |  | 13 |
| Graphic Element |  | 8 |
| Use of Color |  | 6 |
| Audio Element |  | 0 |
| Animated/Moving Element |  | 0 |
| Structure |  | 3 |
| Structured Layout |  | 1 |
| Step-by-Step Element |  | 0 |
| Open Format |  | 1 |
| Legibility |  | 0 |
| Other |  | 0 |
| Meets Expectations |  | 10 |
| Personal Preference/Experience |  | 4 |
| Does Not Meet Expectations |  | 6 |
| Desires |  | 0 |
| Simple Narrative |  | 0 |
| Physical Consent |  | 0 |
| Digital Consent |  | 0 |
| No Abstractions |  | 0 |
| Other |  | 0 |
| Infographic |  | 110 |
| Personal Preference/Experience |  | 11 |
| Does Not Meet Expectations |  | 0 |
| Meets Expectations |  | 12 |
| Desires |  | 2 |
| Text Related Desires |  | 1 |
| No Deception |  | 1 |
| Simple Narrative |  | 0 |
| Physical Consent |  | 0 |
| Digital Consent |  | 0 |
| No Abstractions |  | 0 |
| Other |  | 0 |
| Likes |  | 0 |
| Content Criteria |  | 0 |
| Store/Narrative Element |  | 0 |
| Interplay of Text and Graphics |  | 3 |
| Language Criteria |  | 0 |
| Readability |  | 2 |
| Directness/Conciseness |  | 0 |
| Plain Words |  | 0 |
| Design Criteria |  | 51 |
| Impression |  | 6 |
| Legibility |  | 1 |
| Graphic Elements |  | 16 |
| Use of Color |  | 6 |
| Animated/Moving Element |  | 0 |
| Audio Element |  | 0 |
| Structure |  | 8 |
| Step-by-Step Elements |  | 10 |
| Structured Layout |  | 4 |
| Open Format |  | 0 |
| Relationship Criteria |  | 0 |
| Audience Fit |  | 1 |
| Tone |  | 0 |
| Unconventional Consent Process |  | 3 |
| Other |  | 0 |
| Negative |  | 25 |
| Relationship Criteria |  | 0 |
| Tone |  | 4 |
| Unconventional Consent Process |  | 2 |
| Audience Fit |  | 3 |
| Content Criteria |  | 0 |
| Subject |  | 2 |
| Interplay of Text and Graphics |  | 1 |
| Language Criteria |  | 0 |
| Readability |  | 0 |
| Design Criteria |  | 0 |
| Impression |  | 5 |
| Graphic Element |  | 7 |
| Use of Color |  | 2 |
| Audio Element |  | 0 |
| Animated/Moving Element |  | 0 |
| Structure |  | 0 |
| Structured Layout |  | 0 |
| Step-by-Step Element |  | 0 |
| Open Format |  | 0 |
| Legibility |  | 1 |
| Other |  | 0 |
| Comic |  | 96 |
| Personal Preference/Experience | Preference towards comics or a specific medium, OR no preference towards a medium and towards the content because of the topic | 10 |
| Does Not Meet Expectations |  | 11 |
| Meets Expectations |  | 3 |
| Desires |  | 4 |
| Text Related Desires |  | 3 |
| No Deception |  | 1 |
| Simple Narrative |  | 0 |
| Physical/Paper Consent |  | 0 |
| Digital Consent |  | 0 |
| No Abstractions |  | 0 |
| Other |  | 0 |
| Likes |  | 0 |
| Content Criteria |  | 5 |
| Story/Narrative Element |  | 2 |
| Interplay of Text and Graphics |  | 3 |
| Language Criteria |  | 0 |
| Plain Words |  | 0 |
| Directness |  | 0 |
| Readability |  | 0 |
| Design Criteria |  | 10 |
| Structure |  | 0 |
| Structured Sections/Layout | Includes "step by step flow" of information | 1 |
| Step-by-step Elements (Sequential) |  | 1 |
| Open Format (jump sections) |  | 0 |
| Graphic Elements |  | 7 |
| Audio Element |  | 0 |
| Animated/Moving Elements |  | 0 |
| Use of Color |  | 1 |
| Relationship Criteria |  | 0 |
| Tone |  | 1 |
| Unconvential Consent Process |  | 5 |
| Audience Fit |  | 2 |
| Other |  | 0 |
| Dislikes |  | 45 |
| Language Criteria |  | 0 |
| Readability |  | 4 |
| Content Criteria |  | 1 |
| Story/Narrative Element |  | 0 |
| Interplay of Text and Graphics |  | 1 |
| Design Criteria |  | 16 |
| Legibility |  | 1 |
| Structure |  | 1 |
| Open Format |  | 0 |
| Step-by-Step Element(Sequential) |  | 0 |
| Structured Sections/Layout |  | 0 |
| Impression |  | 9 |
| Graphic Element |  | 5 |
| Audio/Visual Element |  | 0 |
| Use of Color |  | 0 |
| Animated/Moving Elements |  | 0 |
| Relationship Criteria |  | 0 |
| Audience Fit |  | 10 |
| Tone |  | 8 |
| Unconventional Consent Process |  | 6 |
| Other |  | 0 |
| Emotions |  | 247 |
| Neutral |  | 7 |
| Other |  | 7 |
| Disgust |  | 5 |
| Boredom |  | 23 |
| Contempt/Rejection |  | 7 |
| Anger |  | 0 |
| Annoyance |  | 14 |
| Aggressiveness |  | 0 |
| Vigilance/Alertness/Clear |  | 17 |
| Anticipation/Attentive |  | 22 |
| Interest |  | 20 |
| Optimism |  | 2 |
| Joy |  | 8 |
| Serenity/Calmness |  | 17 |
| Love |  | 0 |
| Admiration |  | 2 |
| Trust | Closest to Admiration | 9 |
| Acceptance |  | 22 |
| Submission |  | 0 |
| Fear |  | 0 |
| Apprehension/Anxiety |  | 5 |
| Awe | Awe is a mix of fear and amazement | 0 |
| Amazement |  | 5 |
| Surprise |  | 19 |
| Distraction/Confusion |  | 16 |
| Disapproval |  | 19 |
| Sadness |  | 1 |
| Pensiveness |  | 0 |
| Remorse |  | 0 |
| Consent Desires |  | 24 |
| Consent Management |  | 1 |
| Time/Brevity |  | 1 |
| Personal Engagement |  | 4 |
| Interactive Elements |  | 2 |
| Audio Element |  | 1 |
| Multiple Mediums |  | 3 |
| Tone |  | 1 |
| Testing Understandability |  | 2 |
| Graphic Elements |  | 5 |
| Data Processing |  | 3 |
| Risks |  | 1 |
| Flow |  | 1 |
| Revocation |  | 1 |
| Other |  | 0 |
| Engaging Elements |  | 39 |
| Text |  | 1 |
| Not Applicable |  | 6 |
| Data Processing Context | e.g. if the service "forces" data processing, | 1 |
| Language Criteria |  | 1 |
| Readability |  | 1 |
| Design Criteria |  | 24 |
| Impression |  | 3 |
| Graphic Elements |  | 2 |
| Animated/Moving Elements |  | 1 |
| Highlighting |  | 3 |
| Use of Color |  | 3 |
| Structure |  | 6 |
| Audio Element |  | 2 |
| Headings |  | 4 |
| Enabler |  | 3 |
| Time |  | 3 |
| Consent Management |  | 0 |
| Content Criteria |  | 3 |
| Story/Narrative Elements |  | 2 |
| Interplay of Text and Graphics |  | 1 |
| Expectations for Consent Management |  | 52 |
| One Stop Shop |  | 9 |
| Other |  | 4 |
| Physical |  | 3 |
| Digital |  | 12 |
| Data Controller/Processor Centric Service |  | 0 |
| User Centric Service |  | 0 |
| Management Platform | App, dashboard, etc as a place to control consent | 20 |
| Sending email/letter |  | 4 |
| Clicking a button on a website |  | 0 |
| Other interactive button type | Such as sliders, on/off buttons, etc | 0 |
| Signature |  | 0 |
| Expectations for Withdrawal |  | 39 |
| One Stop Shop |  | 4 |
| Other |  | 0 |
| Physical |  | 2 |
| Digital |  | 7 |
| Data Controller/Processor Centric |  | 0 |
| User-Centric Service |  | 0 |
| Sending email/letter |  | 4 |
| Clicking a button |  | 4 |
| Signature |  | 0 |
| Management Platform |  | 18 |
| Reasoning for Withdrawal |  | 48 |
| Other |  | 2 |
| Consent Used for Other Purposes |  | 10 |
| Lost Trust/Confidence |  | 8 |
| Fear of Being Exploited |  | 11 |
| Data Breach/Leakage |  | 8 |
| Increased Desire for Control |  | 0 |
| Disinterest in Service |  | 9 |
| No benefit |  | 0 |
| Quotable Example |  | 0 |
